# *Helicobacter pylori* Infection and Anemia: The Potential Role of Vitamin C and Vitamin B_12_

**DOI:** 10.3390/molecules31091406

**Published:** 2026-04-24

**Authors:** Joanna Wróblewska, Marcin Wróblewski, Anna Długosz, Lena Pater, Weronika Wróblewska, Carmelo Rizzo, Alina Woźniak

**Affiliations:** 1 Department of Medical Biology and Biochemistry, Faculty of Medicine, Ludwik Rydygier Collegium Medicum in Bydgoszcz, Nicolaus Copernicus University in Toruń, 24 Karłowicza St., 85-092 Bydgoszcz, Poland; joanna.wroblewska@cm.umk.pl (J.W.); al1103@cm.umk.pl (A.W.); 2 Department of Bromatology and Pharmacology, Faculty of Chemical Technology and Engineering, Bydgoszcz University of Science and Technology, 3 Seminaryjna St., 85-326 Bydgoszcz, Poland; 3 Student Scientific Club of Perinatology and Gynecology, Poznan University of Medical Sciences, Collegium Maius, 10 Fredry St., 61-701 Poznań, Poland; 93392@student.ump.edu.pl; 4 Student Scientific Club of Biochemistry and Bioorganic Chemistry, Department of Medical Biology and Biochemistry, Faculty of Medicine, Ludwik Rydygier Collegium Medicum in Bydgoszcz, Nicolaus Copernicus University in Toruń, 24 Karłowicza St., 85-092 Bydgoszcz, Poland; 316714@stud.umk.pl; 5 School of Clinical Nutrition, International Academy of Clinical Nutrition (A.I.N.C.), Via Merulana 13, 00185 Rome, Italy; info@carmelorizzo.it

**Keywords:** *Helicobacter pylori*, anemia, vitamin B12, vitamin C

## Abstract

Anemia is a major global public health problem and is most commonly associated with iron deficiency; however, deficiencies in other micronutrients, including vitamin B_12_, may also contribute to its development. Increasing evidence suggests that *Helicobacter pylori* infection may influence the occurrence of anemia through several mechanisms related to alterations in the gastric environment. Chronic gastric inflammation and increased gastric pH associated with *H. pylori* infection may impair the absorption of non-heme iron and reduce the concentration of vitamin C in gastric juice. Since vitamin C enhances iron bioavailability by reducing ferric iron (Fe^3+^) to the more absorbable ferrous form (Fe^2+^), decreased levels of this vitamin may further limit iron absorption. At the same time, the increase in gastric pH may hinder the release of vitamin B_12_ from food proteins, potentially contributing to disturbances in its absorption. This review aimed to present an integrated overview of the relationships between *H. pylori* infection, alterations in the gastric environment, and mechanisms that may contribute to the development of anemia, including disturbances in vitamin B_12_ absorption, with particular emphasis on the potential role of vitamin C.

## 1. Introduction

Anemia constitutes a significant global public health problem, affecting approximately one-third of the world’s population and being associated with increased morbidity and mortality among affected individuals. Among the various etiologies of anemia, the most common is iron deficiency anemia (IDA), which results from insufficient intake or availability of iron necessary for proper erythropoiesis. As a consequence, hemoglobin (Hb) concentration decreases below the level required to ensure adequate oxygen transport and to meet the physiological needs of the body [1,2]. Oral iron supplementation is the primary treatment for IDA. However, the effectiveness of therapy depends on adequate absorption of this element in the proximal gastrointestinal tract. This process may be limited by concomitant intake of absorption inhibitors (e.g., tea or calcium), coexisting inflammation leading to iron sequestration, intestinal mucosal diseases (e.g., inflammatory bowel disease or celiac disease), *Helicobacter pylori* infection, and impaired gastric acid secretion [1]. Cross-sectional studies conducted in different patient groups, including elderly individuals, children, and adults with dyspeptic symptoms, have demonstrated a significant association between *H. pylori* infection and an increased prevalence of anemia. These findings are largely based on clinical populations rather than population-based studies. The prevalence of anemia among infected individuals ranged from approximately 5% to 29%, depending on the study population and diagnostic criteria [3,4,5].

In the case of *H. pylori* infection, disturbances in iron metabolism may result, among other mechanisms, from decreased gastric acid secretion, which limits the solubility and absorption of dietary iron, and from competition between the bacterium and the host for available iron resources. Furthermore, bacterial strains isolated from patients with IDA may exhibit a greater capacity to take up inorganic iron than strains derived from individuals without iron deficiency [6]. The presence of *H. pylori* infection may also affect iron homeostasis by persistently inflaming the gastric mucosa, thereby disrupting the body’s metabolism of this element [7].

Impaired iron absorption may lead to microcytic anemia, characterized by decreased Hb concentration, low hematocrit, and reduced mean corpuscular volume (MCV). In peripheral blood smears, hypochromic erythrocytes are typically observed, often with a characteristic pencil-shaped morphology [8]. It should be emphasized, however, that *H. pylori* infection may affect the hematopoietic system not only through disturbances in iron metabolism. Chronic gastritis induced by this bacterium may also impair the absorption of vitamin B_12_ (cyanocobalamin). Consequently, in addition to microcytic anemia associated with iron deficiency, some patients may develop macrocytic anemia due to vitamin B_12_ deficiency [9]. Macrocytic anemia is characterized by an increased MCV of >100 fL. In megaloblastic anemia, which most commonly results from vitamin B_12_ or folate deficiency, peripheral blood smear examination reveals macro-ovalocytes and hypersegmented neutrophils. These changes arise from impaired DNA synthesis in erythroid precursor cells, leading to the formation of enlarged red blood cells [10]. Vitamin B_12_ deficiency most commonly manifests as mild to moderate macrocytic anemia; however, in cases of prolonged and severe deficiency, it may also lead to pancytopenia and serious neurological disorders [9].

Vitamin C may enhance iron absorption by acidifying the gastrointestinal environment and facilitating the reduction of poorly absorbable ferric iron (Fe^3+^) to its more readily absorbed ferrous form (Fe^2+^) [1]. However, mechanistic evidence indicates that vitamin C plays an important role in enhancing the absorption of non-heme iron; this effect depends on the dose, the molar ratio of vitamin C to iron, and the presence of meal inhibitors. Therefore, the influence of vitamin C on iron bioavailability may vary depending on the composition of the diet [11]. Regardless of its potential influence on iron absorption, vitamin C also exerts a protective effect on the gastric mucosa, mainly through its antioxidant properties. Ascorbic acid reduces oxidative stress by neutralizing reactive oxygen species (ROS) and inhibiting endogenous *N*-nitrosation and the formation of *N*-nitroso compounds. Furthermore, it contributes to maintaining antioxidant defense systems by regenerating other antioxidants, such as vitamin E and glutathione (GSH). Humans cannot synthesize ascorbic acid endogenously because they lack L-gulonolactone oxidase; therefore, it must be obtained from dietary sources [12].

Vitamin B_12_ plays a crucial role in processes of intensive cellular proliferation, including erythropoiesis. Its deficiency disrupts proper DNA synthesis in hematopoietic precursor cells, leading to the development of characteristic megaloblastic changes in the bone marrow and the occurrence of megaloblastic anemia. Furthermore, vitamin B_12_ deficiency may result in increased levels of homocysteine and methylmalonic acid, which are considered biochemical markers of this deficiency [13].

This study aims to review and analyze current evidence regarding the impact of *H. pylori* infection on the development of anemia, with particular emphasis on changes in the gastric environment, vitamin B_12_ status, and the potential role of vitamin C in modulating iron absorption, protecting the gastric mucosa, and improving the effectiveness of *H. pylori* eradication.

## 2. Methods

**Figure 1:**
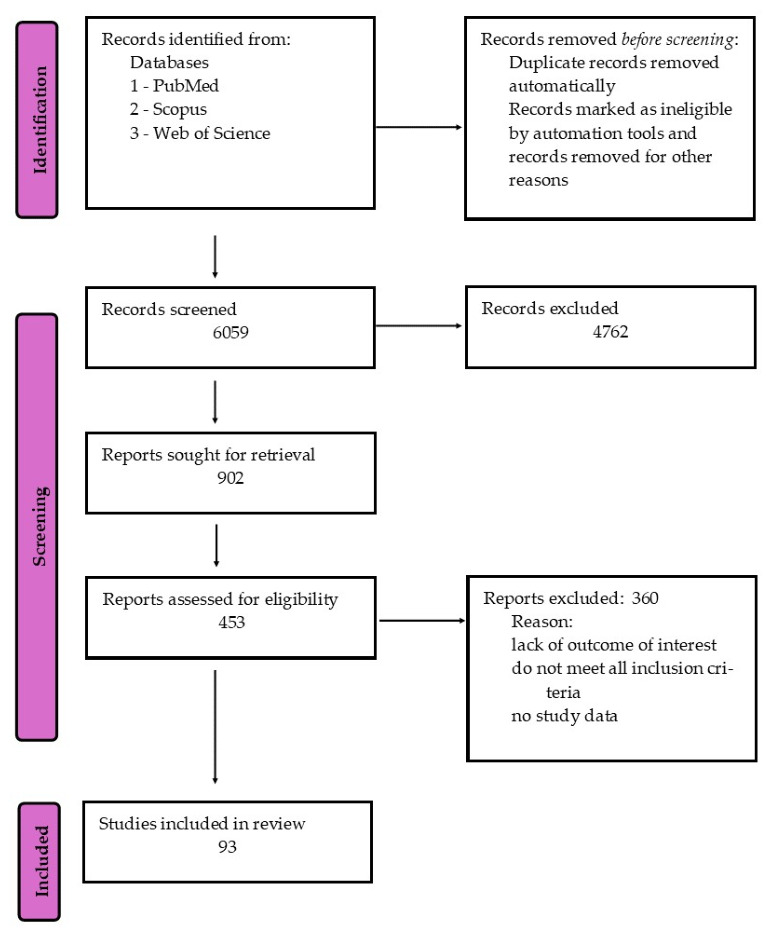
Literature search strategy.

This study was a narrative review aimed at synthesizing current knowledge of the relationship between *H. pylori* infection and anemia, with particular emphasis on the roles of vitamin C and vitamin B_12_ in iron metabolism and gastric physiology. A comprehensive literature search was conducted across the following databases: PubMed, Scopus, and Web of Science. The search included articles published up to March 2026. The following keywords and Medical Subject Headings (MeSH) terms were used in various combinations: “*Helicobacter pylori*”, “anemia” OR “iron deficiency anemia”, “vitamin C” OR “ascorbic acid”, “vitamin B_12_” OR “cobalamin”, “iron absorption”, “gastric pH”, “gastritis”. Boolean operators (AND, OR) were applied to refine the search. There were no restrictions on data collection. No language restrictions were applied during the analysis. However, we tried to select articles from the last 20 years. After searching, we further examined the full texts of the literature to determine eligibility for inclusion in this review. Editorials, conference abstracts, and studies with incomplete or unavailable data were excluded. The methodology discussed enabled the selection of records for this review (Figure 1).

## 3. Vitamin C in the Context of Biochemistry, Nutrition, and Human Health

Vitamin C is an essential, water-soluble micronutrient [14]. It is naturally present in many plant-based foods, particularly in citrus fruits (e.g., lemon and orange), as well as in strawberries, tomatoes, tamarind, amla fruits, and cruciferous vegetables such as brussels sprouts [15]. In plant cells, vitamin C is present in the cytosol, chloroplasts, vacuoles, mitochondria, and the extracellular matrix [14]. Its content in plant tissues depends on the activity of ascorbate biosynthesis and recycling pathways, as well as on the cells’ metabolic status and hydration. In strongly dehydrated structures, such as dry seeds, the oxidized form of vitamin C, dehydroascorbic acid (DHA), predominates [16]. Compared with plant sources, which may contain very high amounts of ascorbic acid (up to approximately 5000 mg/100 g), animal-derived products are characterized by a significantly lower content of this vitamin, usually not exceeding 30–40 mg/100 g [15]. Selected quantitative data regarding vitamin C content in foods, intake recommendations, safety limits, and doses used in experimental and clinical studies are summarized in Table 1 and Table 2. Ascorbic acid is used in the food industry as an antioxidant and anti-browning agent because it protects the sensory and nutritional properties of food. It inhibits enzymatic browning by reducing the *o*-quinones formed during polyphenol oxidase-catalyzed reactions to their original diphenols and by lowering the pH, which limits polyphenol oxidase activity and slows the browning process in plant products [17]. Due to its biochemical properties and widespread presence in plant-derived foods, vitamin C exerts numerous physiological functions in the human body. Vitamin C plays an important role in the differentiation and proper function of immune cells and epithelial barrier cells, supporting barrier integrity and the organism’s immune response [18].

**Figure 2:**
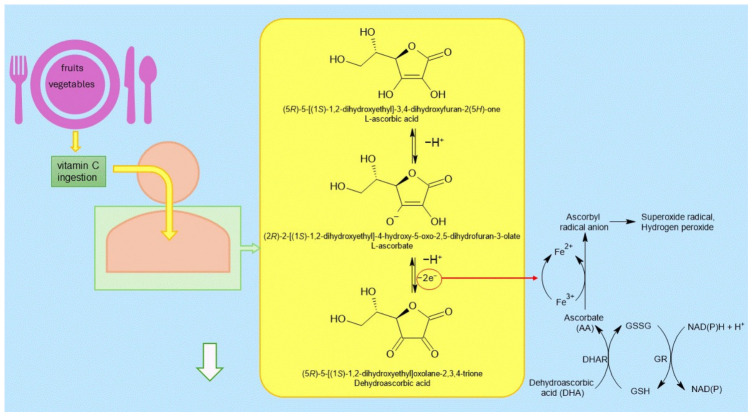
The Foyer–Halliwell–Asada pathway involved in the detoxification of hydrogen peroxide (H_2_O_2_) and maintenance of cellular redox balance. Abbreviations: ascorbate (AA), dehydroascorbic acid (DHA), dehydroascorbate reductase (DHAR), glutathione reductase (GR), reduced glutathione (GSH), oxidized glutathione (GSSG).

Under physiological pH conditions, vitamin C occurs mainly in the form of the ascorbate anion. At pH 7, approximately 99.9% of vitamin C exists as the monoanion; therefore, its biological activity is primarily associated with this form. As an electron donor, ascorbate can undergo two consecutive one-electron oxidation reactions, leading to the formation of DHA and termination of radical chain reactions [14]. The DHA formed can subsequently be reduced to ascorbate by dehydroascorbate reductase using GSH as a reductant. In contrast, glutathione reductase utilizes NADPH to regenerate GSH, thereby maintaining the continuity of the Foyer–Halliwell–Asada pathway (Figure 2) [16]. The Foyer–Halliwell–Asada pathway participates in the removal of hydrogen peroxide (H_2_O_2_) and protects various cellular compartments from oxidative damage [14]. The antioxidant activity of vitamin C is dose-dependent. Experimental studies have shown that administration of vitamin C at a dose of 100 mg/kg body weight per day produces a clear antioxidant effect. The use of a higher dose, 200 mg/kg body weight per day, is associated with a greater protective effect across various tissues. Moreover, supplementation with 200 mg/kg body weight of vitamin C per day increases antioxidant enzyme activity and reduces lipid peroxidation [19]. However, the strong reducing properties of vitamin C may, under certain conditions, promote pro-oxidant reactions. Ascorbate reduces Fe^3+^ ions to Fe^2+^ and Cu^2+^ ions to Cu^+^, which may lead to the formation of ROS, including hydroxyl radicals in Fenton-type reactions [14].

Vitamin C binds only minimally to plasma proteins and circulates mainly in a free form. As a hydrophilic molecule carrying a negative charge at physiological pH, it does not readily penetrate the lipid bilayer, and its passive diffusion is limited and slow. Cellular uptake of ascorbate occurs via specific transporters and can occur via both active transport and facilitated diffusion. The sodium-dependent mechanism is mediated by sodium-ascorbate cotransporters (SVCT), whereas facilitated diffusion occurs via hexose transporters [19]. Two saturable isoforms of sodium-dependent vitamin C transporters (SVCT1 and SVCT2), which differ in their affinity for ascorbate and tissue distribution, participate in the sodium-dependent transport process [20]. The absorption of vitamin C is saturable and depends on the activity of SVCT transporters in the small intestine. At high concentrations, absorption is limited by saturation of these mechanisms and by regulation of transporter expression, indicating a physiological limit to vitamin C absorption [15].

The concentration of vitamin C varies depending on the type of tissue and body fluid, whereas its serum level largely depends on recent dietary intake. The highest concentrations are observed in the adrenal glands (4–10 mM) and in the brain (2–10 mM) [19]. High levels of vitamin C are also found in the gastric mucosa, where ascorbate concentrations may be several times higher than in plasma, reflecting active transport into gastric gland cells [20]. In plasma, vitamin C typically ranges from 40 to 60 μM [19]. No significant differences are observed between vitamin C concentrations in serum and plasma; therefore, these values are considered comparable in clinical practice [18]. During infections, a rapid decrease in vitamin C levels is observed in both plasma and leukocytes [19]. Chronic gastritis is also associated with a significant decrease in the concentration of vitamin C and its reduced form in gastric juice. Under conditions of hypochlorhydria, the level of ascorbic acid may be very low or nearly undetectable. It has been suggested that the reduction in its concentration in gastric juice may result from oxidation of ascorbic acid through reaction with nitrite produced by bacteria [21].

Representative quantitative data on intake recommendations [22,23,24], safety considerations, and selected supplementation regimens are summarized in Table 1 and Table 2. Ascorbic acid acts in the lumen of the upper gastrointestinal tract as a ligand of the common non-heme iron pool, enhancing its absorption by improving the solubility of both naturally occurring dietary iron and iron added to fortified foods [11], such as wheat and maize flour, rice, breakfast cereals, cereal products, milk, and infant foods [25]. Its effect is most pronounced when consumed together with a meal containing iron. From a nutritional perspective, vitamin C is particularly important in diets in which plant-based foods are the main source of iron, as these foods provide non-heme iron with lower bioavailability. The presence of phytates, polyphenols, calcium, and certain plant proteins may limit the absorption of this form of iron. In contrast, ascorbic acid can partially counteract the effects of these inhibitors [11].

Beyond its nutritional role and safety profile, vitamin C has also been investigated as an adjunct to oral iron therapy [18]. Although this approach is biologically plausible, given vitamin C’s role in enhancing iron absorption, clinical studies have not consistently shown improved outcomes [2]. As summarized in selected studies presented in Table 2, pooled analyses and randomized trials suggest that any hematological benefit is generally modest, and the available evidence remains inconclusive. In a pooled analysis of clinical studies, the combination group showed a statistically significant increase in Hb and ferritin levels; however, the mean difference in Hb concentration was small (0.14 g/dL), indicating limited clinical relevance [1]. Similar findings were reported in a randomized clinical trial among adult patients with IDA, in which no significant differences were observed between iron monotherapy and combined supplementation [26]. Although some studies have reported benefits in selected populations [27,28], the current evidence is still insufficient to support a clear clinical advantage of routine vitamin C co-supplementation.

In vitro studies indicate that ascorbic acid may degrade cobalamin, potentially affecting its cellular bioavailability and metabolism. Under experimental conditions, it has been shown that cyanocobalamin, in the presence of ascorbic acid, is converted to hydroxocobalamin and subsequently undergoes further oxidative degradation, leading to the breakdown of the corrin ring [29,30]. At the same time, epidemiological data do not confirm that higher serum ascorbic acid concentrations are associated with lower vitamin B_12_ levels or with clinical indicators of vitamin B_12_ deficiency in adults [31].

**Table 1:** Quantitative summary of vitamin C intake, food content, and safety-related considerations.

Category	Quantitative Information	Comment/Interpretation	Ref.
Plant-derived foods	Up to approximately 5000 mg/100 g	Highest concentrations reported in some plant sources	[15]
Animal-derived foods	Usually ≤ 30–40 mg/100 g	Considerably lower than in plant foods	[15]
Intake sufficient to achieve saturating plasma concentrations	100–200 mg/day	Generally sufficient in healthy individuals	[22]
Recommended daily intake	75 mg/day for women, 90 mg/day for men	According to Polish nutrition standards cited in the manuscript	[23]
Recommended intake in adults	90–120 mg/day	According to the source cited in the manuscript, for professional guidance on vitamin C intake	[24]
Tolerable upper intake level	Approximately 400–2000 mg/day	Depends on age	[24]
Main adverse effects at very high intake	not quantified; mainly transient gastrointestinal symptoms	Diarrhea, nausea, abdominal discomfort	[24]
Additional safety considerations	Not quantified	Possible increased risk of kidney stone formation and enhanced iron absorption, rarely in healthy individuals	[24]

**Table 2:** Summary of vitamin C doses reported in experimental and clinical studies discussed in this review.

Study Context	Vitamin C Dose	Co-Intervention	Main Observation	Ref.
Experimental studies (antioxidant effect)	100 mg/kg body weight/day	None specified	Clear antioxidant effect reported	[19]
Experimental studies (higher-dose effect)	200 mg/kg body weight/day	None specified	Greater protective effect across tissues; increased antioxidant enzyme activity and reduced lipid peroxidation	[19]
Clinical trial in adult patients with iron deficiency anemia	Vitamin C added to oral iron regimen	Ferrous succinate 100 mg	No significant difference in Hb increase or restoration of iron stores versus iron alone	[26]
Randomized trial in young women with iron deficiency	500 mg vitamin C	50 mg iron, 12 weeks	Increased ferritin concentration and more pronounced restoration of iron stores in the later period compared with iron alone	[27]
Study in adolescent girls with anemia	100 mg vitamin C	Ferrous sulfate + folic acid	Higher ferritin concentration at the end of the intervention compared with iron + folic acid alone	[28]

Hb—hemoglobin.

## 4. Vitamin B_12_ in the Context of Biochemistry, Nutrition, and Human Health

Vitamin B_12_ is an essential nutrient for humans, acting as a coenzyme in numerous mitochondrial and cytosolic metabolic pathways. It participates in the tricarboxylic acid cycle and in one-carbon metabolism, including the methionine and folate cycles. It also plays a role in methylation-regulated processes, including modifications of metabolites, DNA, RNA, and proteins [32]. The main dietary sources of vitamin B_12_ are animal-derived products, including meat, fish, eggs, milk, and dairy products [33]. Vitamin B_12_ is synthesized by certain bacteria, such as *Propionibacterium freudenreichii* [34] and Pseudomonas denitrificans [29], and its presence in animal tissues results from its accumulation along the food chain [33]. The recommended daily intake for adults ranges from 2.4 to 2.8 µg, whereas for infants, children, and adolescents it ranges from 0.4 to 2.8 µg depending on age [35]. Assessment of vitamin B_12_ status most commonly relies on measuring its concentration in serum or plasma. Levels below approximately 200–300 pg/mL are generally considered indicative of deficiency [35].

Absorption of vitamin B_12_ is a multistep process that requires proper gastrointestinal tract function. The acidic environment of the stomach, maintained by parietal cells, together with pepsin’s proteolytic action, releases cobalamin from animal-derived proteins in food. Subsequently, cobalamin binds to haptocorrin (R-protein), a glycoprotein secreted in saliva and gastric juices, which stabilizes it in the acidic gastric environment. After entering the duodenum, haptocorrin is degraded by pancreatic proteases, allowing cobalamin to bind to intrinsic factor (IF). The resulting B_12_—IF complex travels to the terminal ileum, where it is recognized by specific receptors on enterocytes and internalized via endocytosis. After degradation of intrinsic factor, vitamin B_12_ is released within the cell and transported into the bloodstream, where it binds to transcobalamin II. In this form, known as holotranscobalamin, it represents the biologically active fraction of vitamin B_12_ available to cells, in which it is converted into its active coenzyme forms: methylcobalamin and 5′-deoxyadenosylcobalamin [9]. Methylcobalamin serves as a cofactor for methionine synthase, which catalyzes the conversion of homocysteine to methionine with the participation of 5-methyltetrahydrofolate. Adenosylcobalamin, in turn, acts as a cofactor for methylmalonyl-CoA mutase, an enzyme involved in metabolic pathways leading to the formation of succinyl-CoA [13].

Excess vitamin B_12_ accumulates mainly in the liver and kidneys. The total amount of this vitamin in the body of healthy adults averages 2–3 mg, of which approximately half is located in the liver. Part of the hepatic stores, in the amount of approximately 0.5–5.0 µg per day, is secreted into the bile [13]. Liver dysfunction may lead to increased plasma cobalamin concentration. In acute hepatitis, elevated vitamin B_12_ levels are observed in approximately 25–40% of patients, which is associated with hepatocyte damage and the release of stored cobalamin into the circulation. Elevated vitamin B_12_ concentrations may also occur in liver cirrhosis, where their magnitude correlates with disease severity. Increased plasma cobalamin levels are also observed in more than half of patients with hepatocellular carcinoma and in approximately 30–40% of patients with metastatic liver disease [36].

Vitamin B_12_ deficiency is associated with elevated homocysteine and methylmalonic acid levels, which are used as biochemical markers of deficiency [13]. Elevated homocysteine levels contribute to increased oxidative stress [37]. Epidemiological studies have shown that the intake of certain antioxidant vitamins, including vitamin C, may be inversely associated with plasma homocysteine concentrations. Higher dietary vitamin C intake was associated with a lower prevalence of hyperhomocysteinemia among middle-aged and older adults with hypertension [38]. Vitamin B_12_ deficiency may lead to the development of megaloblastic anemia [35]; however, not all individuals with reduced levels of this vitamin develop overt anemia. In a hospital-based observational study of a pediatric population, the prevalence of megaloblastic anemia due to vitamin B_12_ deficiency was reported to be 15.6%, highlighting the significant role of this deficiency in the etiology of anemia in children [39]. In another pediatric study, megaloblastic anemia accounted for 28.6% of all anemia cases, and vitamin B_12_ deficiency was identified in 78% of children with this diagnosis, indicating a strong association between reduced vitamin B_12_ levels and the development of megaloblastic changes in the hematopoietic system [40]. The risk of vitamin B_12_ deficiency is increased in certain populations. Individuals following vegetarian diets, especially vegan diets, are at risk of inadequate intake of this vitamin, as well as infants born to mothers with low consumption of animal-derived products [35]. In addition to inadequate dietary intake, impaired absorption plays an important role in the pathogenesis of vitamin B_12_ deficiency. The most common causes include atrophic gastritis, pernicious anemia, inflammatory bowel diseases, and *H. pylori* infection. These conditions may lead to reduced secretion of hydrochloric acid and intrinsic factor or damage to the terminal ileum, thereby impairing cobalamin absorption and promoting the development of deficiency [9].

## 5. *Helicobacter pylori* as a Potential Foodborne Pathogen

*H. pylori* is a Gram-negative bacterium classified as a Group I carcinogenic pathogen by the International Agency for Research on Cancer, and chronic infection with this microorganism constitutes an important risk factor for the development of gastric cancer [41]. This bacterium is responsible for the development of chronic gastritis, particularly in the pyloric region, as well as gastric and duodenal ulcer disease. Long-term infection may lead to gastric mucosal atrophy and the development of precancerous lesions [12,42]. It should be emphasized, however, that *H. pylori* may also occur as a component of the oral and gastric microbiota in individuals without symptoms of inflammation. In such cases, its presence is not always directly associated with the initiation of gastric cancer but may rather be linked to its recurrence or chronic course. Moreover, colonization of the stomach by this bacterium may influence the composition of the gastric microbiota [43]. It is estimated that *H. pylori* infects approximately half of the global population; however, in many individuals, the infection remains asymptomatic and does not lead to complications. The prevalence of infection is higher in regions with lower socioeconomic status and in overcrowded conditions. Symptoms that may accompany infection include nausea, bloating, a feeling of fullness, and abdominal pain [44].

Transmission of *H. pylori* occurs mainly through the fecal–oral and oral–oral routes. Infection may occur through direct contact with a carrier of the bacterium or through the consumption of contaminated food or beverages [44,45]. The ability of *H. pylori* to form biofilm on the surfaces of water supply system components, such as taps and pipes, has also been demonstrated, suggesting that water systems may represent a potential environmental source of infection [46]. The bacterium can survive in food products such as milk, meat (e.g., beef, lamb, and chicken), vegetables, and ready-to-eat foods (e.g., dry-fermented sausages) [47]. Due to the low infectious dose of *H. pylori*, even a small number of bacteria present in food may pose a potential health risk to consumers [47]. Increasing evidence suggests that vegetables may be a major source of *H. pylori* infection, surpassing the significance of other foods such as milk or meat. Contamination of vegetables may result from contact with irrigation water or contaminated water used for washing. Raw vegetables are particularly susceptible to bacterial contamination [48]. *H. pylori* can survive for a limited time in various food products, including vegetables and dairy products. In vegetables, the survival time of the bacterium is usually shorter than in some dairy products. It has been demonstrated that *H. pylori* may survive for up to 6 days in spinach stored at room temperature and up to 120 h in raw carrots stored at 8 °C [45]. In the case of milk, the bacterium may persist for a longer period. In fresh milk, the survival of *H. pylori* may reach up to 10 days at 4 °C and approximately 3 days at 25 °C. In pasteurized milk, the bacterium may survive for up to 9 days, whereas in UHT milk it may persist for up to 12 days when stored at 4 °C [45]. In fermented and processed dairy products, the bacterium has also been reported; however, its survival in these products is significantly shorter than in fresh or pasteurized milk [45].

Prevention of *H. pylori* infection involves limiting contact with the bacteria through proper hygiene and appropriate handling of food and water. A fundamental element of prevention is thorough hand washing before preparing and consuming meals, as well as avoiding the sharing of utensils and dishes, which reduces the risk of bacterial transmission between individuals [48]. Due to the possible presence of *H. pylori* in some animal-derived products, particularly raw milk, consumption of unpasteurized dairy products may represent a potential source of infection [45]. To reduce the risk of infection, vegetables should be thoroughly washed in clean water and, whenever possible, subjected to thermal processing [48]. Equally important is the quality of water used for drinking and food preparation. It should come from a safe source, and if there are any doubts, it should be boiled or treated appropriately [48]. Refrigeration or freezing of food does not eliminate *H. pylori*; it only allows the bacterium to survive for a limited period. It has been demonstrated, however, that bacterial growth is limited by acidic environments and the presence of organic acids produced during lactic acid fermentation, which may inhibit its growth in some food products [47]. Public education on food and water hygiene is crucial, especially in regions with poor sanitation standards where infections occur most frequently. Effective implementation of these measures may significantly reduce the number of new *H. pylori* infections [48].

## 6. Selected Virulence Factors of *Helicobacter pylori* in the Pathogenesis of Infection

### 6.1. Modulation of Host Immune Response

The ability of *H. pylori* to maintain chronic infection results from its capacity to balance stimulation of the host immune response with survival in an inflammatory environment, which is facilitated by specific virulence factors. One mechanism involves the bacterium’s ability to evade recognition by pattern recognition receptors, which identify pathogen-associated molecular patterns and initiate signaling cascades that lead to inflammatory responses and the elimination of microorganisms. Modification of lipopolysaccharide (LPS) and flagellin by the bacterium reduces its recognition by Toll-like receptors [49]. Additionally, *H. pylori* can disrupt the phagocytic functions of immune cells. These mechanisms may depend on the type IV secretion system encoded by the cag pathogenicity island. At the same time, the vacuolating cytotoxin (VacA) plays an important role in inhibiting phagosome maturation and fusion with lysosomes, thereby impairing macrophage-mediated intracellular bacterial destruction [50,51].

*H. pylori* produces neutrophil-activating protein (HP-NAP), which enhances the inflammatory response by stimulating phagocyte chemotaxis and inducing the generation of ROS. At the same time, the bacterium protects itself against their effects through detoxifying enzymes such as catalase and superoxide dismutase. Furthermore, it disrupts neutrophil function and NADPH oxidase activity, thereby limiting their ability to eliminate microorganisms. *H. pylori* also affects nitric oxide (NO) metabolism in macrophages. One of the host antimicrobial defense mechanisms is the production of NO by inducible nitric oxide synthase (iNOS), whose expression may be activated during *H. pylori* infection. Despite activating this mechanism, *H. pylori* employs strategies that limit nitric oxide activity, primarily through its own arginase, which consumes L-arginine, and by inducing arginase II in host macrophages. This leads to reduced iNOS expression and decreased NO production [49].

### 6.2. Mechanisms Supporting Survival and Colonization in the Gastric Environment

An important factor enabling long-term colonization of the stomach is also the ability of *H. pylori* to survive in a highly acidic environment. The bacterium produces large amounts of urease, an enzyme that catalyzes the hydrolysis of urea into ammonia and carbon dioxide. In addition to its widely recognized role as a key virulence factor that enables survival in acidic conditions, urease may also be a potential target for non-antibiotic therapeutic strategies. Vitamin C has been shown to interfere with urease activity by reducing the nickel ions present in its active center, which are essential for enzymatic function. This effect may lead to decreased enzymatic activity and reduced ability of *H. pylori* to locally neutralize gastric acidity, thereby potentially limiting bacterial survival and colonization [52]. Survival of *H. pylori* in the stomach is further facilitated by its ability to localize beneath the gastric mucus layer, where the pH is higher than in the gastric lumen, creating a more favorable microenvironment for bacterial persistence. The spiral shape of the bacterial cell and the presence of polar flagella enable the bacterium to move actively within the mucus layer and colonize the surface of epithelial cells. In addition, the bacterium exhibits chemotaxis, allowing it to migrate toward more favorable environmental conditions, such as regions with higher pH [53]. Bacterial mimicry involves structural modifications of the bacterial cell that hinder its recognition by the host immune system. During transition into a dormant state, *H. pylori* changes its morphology from a spiral form to a coccoid form corresponding to the viable-but-non-culturable (VBNC) state. The coccoid form is characterized by an inability to grow on standard culture media, while maintaining cellular integrity and limited metabolic activity. This transition may be induced by factors such as nutrient deficiency, temperature changes, elevated pH, limited CO_2_ availability, and exposure to antimicrobial agents, such as antibiotics, proton pump inhibitors (PPIs), or metabolites produced by bacteria of the genera *Lactobacillus* and *Streptococcus* [54,55]. Despite reduced urease activity and slowed metabolism, bacteria in the dormant state retain their infectivity and expression of virulence factors, which may contribute to the persistence of chronic infection [54]. An important factor that may contribute to the persistence of *H. pylori* is its ability to form a biofilm. This structure promotes increased tolerance of the bacterium to environmental factors, the host immune response, and antimicrobial agents, which is associated with the chronic nature of infection characteristic of this pathogen. The process of biofilm formation is linked to changes in the expression of numerous genes, including those involved in stress response (e.g., *hspR*, *hrcA*, *recR*) and those associated with cell envelope structure. In biofilm conditions, increased expression of genes encoding components of the flagellar apparatus (e.g., *flaB*, *flgE*, *flgK*) is also observed; importantly, beyond their motility function, flagella may play a significant structural role, contributing to the organization and stability of the biofilm [56]. *H. pylori* biofilm may occur in the human body, primarily within the gastric mucosa. The presence of *H. pylori* has also been detected in the oral cavity, including dental plaque and gingival pockets. Multispecies biofilm present in the oral cavity may play a role in the pathogenesis of reinfection with this microorganism [46].

### 6.3. Iron Metabolism, Oxidative Stress, and Potential Involvement in Ferroptosis

**Figure 3:**
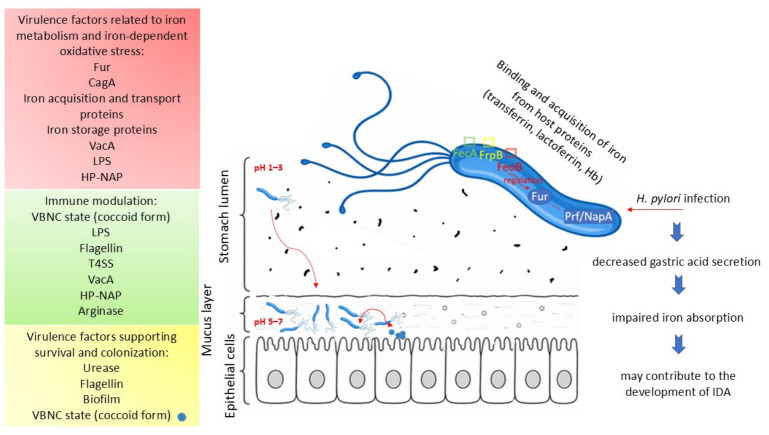
Virulence factors of *Helicobacter pylori.* CagA—cytotoxin-associated gene A; FecA—ferric citrate outer membrane receptor; FeoB—ferrous iron (Fe^2+^) transporter B; FrpB—outer membrane receptor; Fur—ferric uptake regulator; HP-NAP—*Helicobacter pylori* neutrophil-activating protein; Hb—hemoglobin; IDA—Iron-deficiency anemia; LPS—lipopolysaccharide; Pfr—ferritin; VacA—vacuolating cytotoxin A; VBNC—viable-but-non-culturable.

Certain virulence factors of *H. pylori* may influence ferroptosis by modulating iron metabolism, oxidative stress, and host cell antioxidant systems. Host cellular iron homeostasis is influenced by cytotoxin-associated gene A (CagA), which increases iron uptake through transferrin endocytosis and alters intracellular iron distribution, including increased lysosomal iron levels and changes in the cytoplasmic iron pool. Factors influencing ROS production include VacA, HP-NAP, and LPS. VacA increases ROS levels in gastric epithelial cells and disrupts GSH metabolism, thereby weakening cellular antioxidant capacity. Neutrophil-activating protein binds Fe^2+^ ions and participates in their oxidation reactions, leading to the generation of ROS. LPS of *H. pylori* may increase ROS production and influence the expression of glutathione peroxidase 4; however, its direct role in regulating ferroptosis has not yet been clearly established. The metabolism of GSH and cellular antioxidant mechanisms are also influenced by VacA, gamma-glutamyl transpeptidase (gGT), and outer inflammatory protein A. VacA disrupts GSH metabolism in gastric epithelial cells. In contrast, gGT participates in the degradation of GSH into glutamate and cysteinylglycine, thereby reducing intracellular GSH levels. Outer inflammatory protein A decreases the expression of the cystine/glutamate antiporter, which plays a key role in GSH synthesis; however, its direct impact on ferroptosis requires further investigation [41]. It is worth emphasizing that *H. pylori* can bind and acquire iron from host proteins such as transferrin, lactoferrin, and Hb, suggesting the presence of specific mechanisms for their utilization. Importantly, *H. pylori* exhibits a distinct preference for iron-free forms (apo-transferrin and apo-lactoferrin), which distinguishes it from many other pathogenic bacteria that preferentially utilize iron-saturated forms. This mechanism may limit bacterial growth within the host environment while simultaneously promoting its persistence in tissues during chronic colonization [6]. The Ferric Uptake Regulator (Fur) protein plays a key role in regulating iron homeostasis in *H. pylori*, controlling the expression of genes involved in iron uptake and storage in response to changes in iron availability [57,58]. This protein also functions as a global regulator of gene expression, participating in the regulation of bacterial adaptive mechanisms to the gastric environment, including responses to low pH and ROS [58]. Although Fur is best characterized in bacteria as a regulator of iron transport, in *H. pylori* it also controls iron storage, modulates urease expression in response to nickel, and is important for bacterial resistance to acidic conditions [57]. This bacterium possesses a range of proteins associated with iron metabolism, including potential components of iron transport systems, such as outer membrane receptors encoded by the *fecA* and *frpB* genes, periplasmic iron-binding proteins (*ceuE*), components of an ABC transporter (*fecD* and *fecE*), and the Fe^2+^ transporter (*feoB*), as well as proteins involved in iron storage, such as Pfr and NapA [57]. The impact of these factors on iron homeostasis and oxidative stress may, in turn, indirectly affect host iron metabolism. In the course of chronic *H. pylori* infection, these alterations may promote the development of iron deficiency and anemia. A schematic overview of the key virulence factors of *H. pylori* involved in host immune modulation, survival in the gastric environment, and iron metabolism is presented in Figure 3.

## 7. *Helicobacter pylori* and Vitamin C

By creating a local microenvironment, *H. pylori* induces inflammation in the gastric mucosa, leading to neutrophil and macrophage infiltration and increased ROS production. Excessive ROS production promotes oxidative damage to DNA and the oxidation of lipids and proteins [49,59]. Vitamin C, as a potent antioxidant, can neutralize ROS and limit lipid peroxidation [12]. Beyond its antioxidant properties, vitamin C also supports immune function during *H. pylori* infection. It has been shown that immune cells actively accumulate vitamin C, with intracellular concentrations increasing significantly during inflammatory responses. This process enhances the activity of immune cells involved in pathogen clearance, including phagocytes. Consequently, adequate vitamin C levels may contribute to a more effective immune response against *H. pylori* infection [52]. The ability of *H. pylori* to penetrate the gastric mucosa and establish persistent infection may also be influenced by host structural factors. Vitamin C plays an important role in collagen synthesis, particularly type IV collagen, which is a major component of the lamina propria underlying the gastric epithelium. Adequate collagen integrity may act as a physical barrier limiting bacterial infiltration into deeper tissue layers. In contrast, reduced vitamin C levels may weaken this barrier, facilitating bacterial invasion and contributing to the progression of infection [52]. *H. pylori* infection may reduce gastric and systemic vitamin C levels, thereby impairing antioxidant defenses and contributing to increased oxidative stress in the gastric mucosa [60]. Furthermore, vitamin C has been shown to reduce the transition of *H. pylori* into dormant states, such as VBNC and persister cells, and to promote the recovery of VBNC cells, accompanied by an increased proportion of spiral form [54].

**Figure 4:**
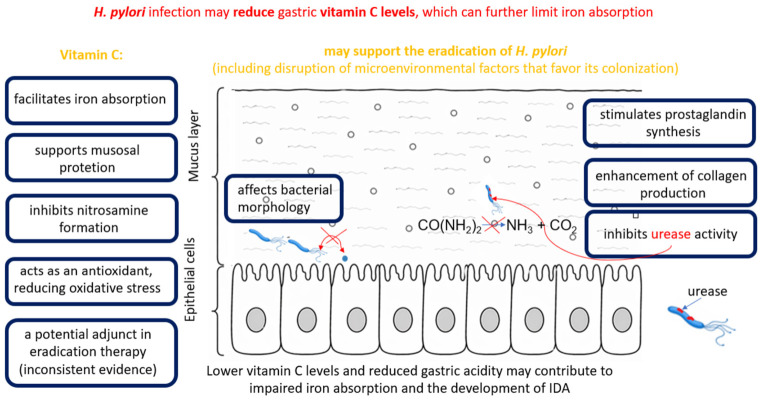
Potential role of vitamin C in *Helicobacter pylori* infection and associated gastric alterations. IDA—Iron-deficiency anemia.

The penetration of antibiotics into tissues increases as inflammation decreases, and the efficacy of a given antibiotic improves as *H. pylori* colonization decreases [59]. Disruption of the microenvironment that favors *H. pylori* colonization by vitamin C may further enhance the effectiveness of antibacterial treatment [59], as it may also exert direct antibacterial effects through multiple mechanisms, including inhibition of urease activity, stimulation of prostaglandin synthesis, and enhancement of collagen production within the gastric mucosa. Increased prostaglandin synthesis contributes to mucosal protection by promoting mucus and bicarbonate secretion, thereby strengthening the gastric barrier against bacterial damage [52]. The effects of vitamin C on *H. pylori* and the gastric microenvironment are illustrated in Figure 4. For this reason, attempts have been made to evaluate the effect of vitamin C supplementation on the efficacy of *H. pylori* eradication. However, it is important to emphasize that many studies assessing the impact of antioxidants on *H. pylori* eradication have examined combined supplementation with vitamin C and vitamin E, given their complementary antioxidant effects. Vitamin C regenerates oxidized vitamin E formed during the neutralization of ROS, whereas vitamin E acts as an antioxidant that buffers ROS [61]. Selected results of these studies are presented in Table 3. Available studies indicate that the effect of vitamin C supplementation on *H. pylori* eradication efficacy is inconsistent and depends on the treatment regimen, dosage, and concurrent supplementation with other antioxidants. An interventional study also demonstrated that, in asymptomatic adults, *H. pylori* infection does not significantly affect vitamin C bioavailability, as assessed by changes in plasma vitamin C concentration following supplementation [60].

The interpretation of the included studies is limited by several factors, including differences in eradication regimens, small sample sizes [65], and the inclusion of specific patient subgroups [67]. This is reflected in inconsistent outcomes, with some studies reporting no benefit or even reduced eradication efficacy following vitamin C supplementation [65,67], while others demonstrate a significant improvement in treatment success [64]. Furthermore, interpretation is complicated by the fact that in several studies vitamin C was administered in combination with vitamin E, making it difficult to distinguish the independent effect of vitamin C from the potential synergistic action of combined antioxidant supplementation. Therefore, the available evidence does not allow for a clear conclusion regarding the specific contribution of vitamin C to *H. pylori* eradication.

Impaired iron absorption, associated with reduced gastric acidity and decreased ascorbic acid levels in the course of *H. pylori*-induced gastritis, particularly in its atrophic form, has been suggested as one of the proposed mechanisms underlying the development of IDA in adults [68]. The absorption of non-heme iron, which is present in the diet in the ferric form, requires its reduction to the ferrous form in the acidic environment of the stomach. This reaction is facilitated by ascorbic acid, which is considered one of the most important factors enhancing iron bioavailability [63]. However, it has not been fully elucidated whether alterations in gastric acidity and decreased ascorbic acid levels may constitute a mechanism linking *H. pylori* infection with the development of IDA [69]. In the study by Annibale et al. [69], it was demonstrated that patients with gastritis induced by *H. pylori* and unexplained IDA exhibited a concomitant increase in intragastric pH and a decrease in gastric juice ascorbic acid concentration, which may contribute to impaired iron absorption.

In the context of these observations, attention has also been drawn to the potential role of vitamin C in protecting the gastric mucosa. In an intervention study conducted by Sasazuki et al. [70], long-term vitamin C supplementation was shown to affect parameters of gastric mucosal status, including the pepsinogen I/II ratio, a marker of atrophic changes in the stomach. The authors suggest that vitamin C may play a protective role in the progression of gastric mucosal atrophy, although its effect on *H. pylori* infection appears to be limited. At the same time, *H. pylori* infection and the associated atrophic changes in the gastric mucosa may be linked to alterations in gastric function, including changes in acid secretion. In the study by Sung et al. [71], it was demonstrated that gastric juice pH was significantly higher in patients infected with *H. pylori* than in uninfected individuals. Moreover, higher pH values were more frequently associated with atrophic gastritis and intestinal metaplasia in the gastric body, suggesting reduced gastric acid secretion during the progression of mucosal changes. At the same time, it has been suggested that changes in gastric acid secretion and the associated alterations in pH may influence the concentration of vitamin C in gastric juice [72]. The effect of *H. pylori* infection on vitamin C levels in the stomach remains inconclusive in the literature. Phull et al. [73] analyzed vitamin C concentrations in the gastric and duodenal mucosa of patients with *H. pylori* infection and in uninfected individuals. The authors found no significant differences in vitamin C levels in the examined tissues between the two groups, suggesting that *H. pylori* infection does not necessarily reduce vitamin C levels in the gastrointestinal mucosa. Different results were obtained by Zhang et al. [74], who demonstrated that *H. pylori* infection is associated with a significant reduction in ascorbic acid concentrations in both gastric juice and the gastric mucosa. Furthermore, the authors observed that in patients infected with CagA-positive *H. pylori* strains, gastric juice vitamin C levels were even lower than in those infected with CagA-negative strains. Similar relationships were reported by Capurso et al. [75], who demonstrated a stepwise decrease in ascorbic acid concentrations in gastric juice from healthy individuals to patients with non-atrophic gastritis associated with *H. pylori*, and then to patients with atrophic body gastritis. The authors also found a significant negative correlation between ascorbic acid levels in gastric juice and intragastric pH, suggesting that higher gastric pH may reduce ascorbic acid concentration. These findings indicate that an increase in gastric juice pH, observed during inflammation and mucosal atrophy, may play an important role in reducing vitamin C levels.

In gastric fluid, vitamin C reduces the concentration of N-nitrosamines [61], which are compounds with well-established mutagenic and carcinogenic properties [76,77]. They form in the stomach as a result of the reaction between nitrites and amines or amides, particularly under acidic conditions. Vitamin C inhibits the nitrosation process by blocking the formation of nitrosating agents, and daily doses ranging from approximately 9 mg to 1000 mg have been reported to reduce nitrosamine formation by 20–100% [77].

## 8. Impact of *Helicobacter pylori* Infection on Vitamin B_12_ Status and Hematological Parameters

*H. pylori* infection may affect the gastric environment and be associated with an increase in gastric juice pH [71]. This bacterium may affect gastric acid secretion and lead to the development of atrophic gastritis, which can impair vitamin B_12_ absorption [78]. A less acidic environment may impair the release of vitamin B_12_ from food proteins [79]. The inability to absorb protein-bound cobalamin, despite the preserved ability to absorb free cobalamin, is defined as food-cobalamin malabsorption. Cobalamin malabsorption is common in older adults due to achlorhydria and hypochlorhydria, bacterial overgrowth, and decreased synthesis and secretion of intrinsic factor [80]. The complexity of the mechanisms underlying cobalamin malabsorption is further supported by the findings of Cohen et al. [81], who demonstrated that malabsorption of protein-bound cobalamin may occur even in the absence of atrophic gastritis and achlorhydria, suggesting heterogeneity in the histological and functional changes of the stomach in affected patients.

Clinical studies indicate that *H. pylori* eradication therapy is associated with increased serum vitamin B_12_ levels, with a greater rise observed in patients who successfully eradicate the infection. In the study by Serin et al. [82], the reduction in *H. pylori* density after therapy was more pronounced than the improvement in gastric mucosal inflammation and neutrophil activity. At the same time, serum vitamin B_12_ levels increased within a relatively short period, as early as 2–3 months after treatment. Kaptan et al. [83] suggest a clinically meaningful and potentially reversible association. The study demonstrated that successful eradication of *H. pylori* may lead to normalization of serum vitamin B_12_ levels and improvement in hematological parameters without the need for cobalamin supplementation, whereas patients with persistent infection often required replacement therapy. Furthermore, recurrence of infection was associated with a subsequent decline in vitamin B_12_ levels, with re-treatment leading to renewed improvement, indicating an infection-dependent and reversible mechanism.

**Figure 5:**
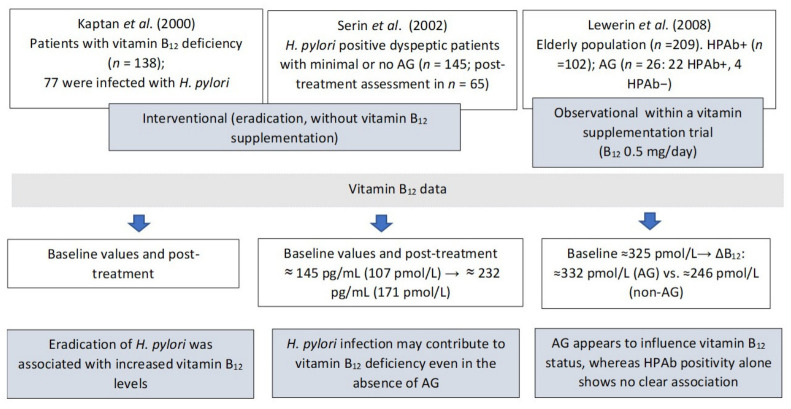
Comparison of study designs and reporting of vitamin B_12_ status. Δ (delta) represents the change in serum vitamin B_12_ concentration (post-treatment minus baseline); AG—atrophic gastritis; HPAb—antibodies against *Helicobacter pylori*; *n*—number of patients [82,83,84].

Moreover, some patients with high *H. pylori* colonization density exhibited normal vitamin B_12_ levels, which may be partially explained by higher dietary intake of the vitamin, for example, through multivitamin supplements or foods rich in cobalamin. The heterogeneity in study design and reporting of vitamin B_12_ measurements across these studies is summarized in Figure 5. Interventional data suggest that eradication of *H. pylori* may be associated with improvements in hematological parameters, including normalization of MCV and increases in hematocrit. These findings indicate a possible relationship between H. pylori infection, vitamin B_12_ deficiency, and alterations in red blood cell indices; however, a causal link has not been definitively established [83]. In contrast, population-based data in elderly individuals indicate that atrophic gastritis, rather than *H. pylori* seropositivity alone, may play a more prominent role in determining vitamin B_12_ status and related hematological parameters. In this context, atrophic gastritis has been associated with lower hemoglobin levels. Changes in MCV have been observed only in small subgroups of patients, particularly those with atrophic gastritis without detectable *H. pylori* antibodies, and should be interpreted with caution [84]. Furthermore, evidence from patients with minimal or no gastric atrophy indicates that *H. pylori* infection may affect vitamin B_12_ status and hematological parameters even in the absence of significant gastric atrophy. In this study, hemoglobin levels increased following treatment, whereas MCV remained unchanged [82].

*H. pylori* infection may be associated with the presence of antibodies against gastric parietal cells in some patients [81]. Parietal cells produce intrinsic factor, which is essential for the proper absorption of vitamin B_12_. However, it should be emphasized that *H. pylori* infection is not a direct cause of pernicious anemia, which is primarily an autoimmune condition, although the infection may indirectly contribute to its development [44]. It has also been suggested that infection with this bacterium may be associated with iron deficiency anemia, particularly in cases of unexplained etiology, which may be explained, among other factors, by impaired iron absorption and chronic inflammation of the gastric mucosa [7,85]. Iron deficiency may influence the course of *H. pylori* infection, as under conditions of limited iron availability, the bacterium increases the expression of virulence factors such as CagA [41]. It is possible that iron deficiency, by increasing *H. pylori* pathogenicity, may indirectly exacerbate nutrient malabsorption.

## 9. Treatment of *Helicobacter pylori* Infection and Iron Metabolism

Treatment of *H. pylori* infection is challenging, mainly because of the bacterium’s increasing antibiotic resistance. Consequently, eradication regimens have evolved and are currently based on combination therapy. The most commonly used treatment includes a PPI, such as omeprazole, lansoprazole, or pantoprazole, in combination with antibiotics such as amoxicillin, clarithromycin, or tetracycline, as well as nitroimidazole agents, such as metronidazole or tinidazole [52,86,87]. In cases of failure of first-line therapy, bismuth-based quadruple therapy is used, consisting of a proton pump inhibitor in combination with a bismuth salt, metronidazole, and tetracycline [52]. PPIs reduce gastric acidity, thereby improving the bioavailability of antibiotics in the gastric mucosa [86]. At the same time, long-term use of PPIs may affect the absorption of certain nutrients, such as vitamin B_12_, vitamin C, and iron [88]. In the study by Mumtaz et al. [89], long-term PPI therapy was associated with an increased risk of vitamin B_12_ deficiency. However, the results remain inconclusive, as Losurdo et al. [85] found no significant difference in vitamin B_12_ levels between patients using PPIs and the control group. A decrease in gastric juice vitamin C concentration during proton pump inhibitor therapy is associated with a reduced proportion of its active form, ascorbic acid. This phenomenon affects nitrite metabolism in gastric juice, potentially increasing its concentration in the gastric environment [88]. Long-term use of PPIs may be associated with an increased risk of anemia, likely due to reduced iron absorption resulting from decreased gastric acidity [90,91]. Observational studies have also demonstrated a decrease in hematological parameters, such as Hb and hematocrit, in patients receiving long-term PPI therapy [90]. However, the results regarding the impact of PPIs on iron metabolism remain inconclusive. In the study by Losurdo et al. [92], no significant differences in serum iron or ferritin levels were observed between PPI users and the control group.

Iron availability plays a key role in the pathogenicity and colonization ability of *H. pylori* in the gastric mucosa [58]. It has been shown that the addition of ferrous sulfate to the culture medium led to a marked increase in bacterial growth and a shortened lag phase in cultures of various *H. pylori* strains [93]. The cornerstone of IDA treatment is iron supplementation, aimed at restoring normal Hb levels and replenishing iron stores. Numerous nonheme iron supplements are available, with ferrous sulfate and ferric succinate being the most commonly used [26]. Therefore, eradication of *H. pylori* may be considered before or alongside iron supplementation, particularly in selected cases. In a study conducted among children, the hematological response to iron supplementation was significantly weaker in patients infected with *H. pylori* compared with uninfected children, despite similar baseline Hb and ferritin levels [94]. The combination of iron supplementation and *H. pylori* eradication therapy resulted in greater improvement in hematological parameters than iron supplementation alone [95]. Eradication of *H. pylori* combined with iron supplementation increased functional iron, whereas iron supplementation alone increased iron stores [96]. Although studies assessing the hematological response to iron supplementation in patients with *H. pylori* infection have reported a reduced treatment response compared with uninfected individuals and greater improvement with combined therapy [94,95,96], another study by Sarker et al. [97] suggests that iron therapy alone can significantly improve iron status, even in the presence of *H. pylori* infection.

In an animal model, *H. pylori* infection in gerbils was associated with the development of iron-deficiency anemia, as reflected by decreased Hb, MCV, and serum ferritin levels. Importantly, both the incidence and severity of anemia were influenced by dietary composition, with high-salt and low-iron diets exacerbating *H. pylori*-induced iron deficiency anemia, suggesting that nutritional factors may modulate the hematological consequences of infection [98]. Dietary factors may also modulate the clinical consequences of *H. pylori* infection by influencing the gastric environment and micronutrient bioavailability [98]. In particular, vitamin C-rich foods may support the absorption of non-heme iron [11], whereas diets low in bioavailable iron may further aggravate iron deficiency in susceptible individuals [98]. In addition, because gastritis caused by *H. pylori*, altered acid secretion, and increased gastric pH may impair the release of vitamin B_12_ from food proteins [71,78,79], dietary composition may influence the functional availability of this vitamin. Taken together, these observations suggest that dietary modulation may be considered a supportive adjunct to standard therapy, although its clinical value requires further investigation.

## 10. Conclusions

Anemia remains a significant global public health problem, and one of its most common forms is iron deficiency anemia, resulting from insufficient intake or impaired absorption of this element. One factor that may affect iron homeostasis is *H. pylori* infection, which, through alterations in the gastric environment, chronic mucosal inflammation, and bacterial competition for available iron, may reduce its bioavailability and absorption. These observations indicate that *H. pylori* infection should be considered in the diagnostic work-up of patients with unexplained or refractory iron deficiency anemia, particularly when the response to standard iron supplementation is suboptimal.

*H. pylori* infection may also lead to impaired vitamin B_12_ absorption due to changes in the gastric environment, including increased gastric pH and reduced release of cobalamin from dietary proteins. An important role in iron metabolism is also played by vitamin C. Vitamin C is a relatively strong organic acid (pKa ≈ 4.1); however, it does not significantly affect gastric acidity. The stomach environment is already highly acidic (pH approximately 1–2) due to the secretion of hydrochloric acid by parietal cells. The main role of vitamin C in this context is to enhance iron bioavailability by reducing Fe^3+^ to the more absorbable Fe^2+^ form. Although vitamin C is known to interact chemically with vitamin B_12_, current evidence does not confirm a significant effect on cobalamin bioavailability in the gastrointestinal tract. At the same time, available clinical data suggest that routine vitamin C supplementation does not consistently translate into improved hematological outcomes, indicating that its therapeutic role remains uncertain.

*H. pylori* infection may affect the availability of certain micronutrients and vitamins through alterations in the gastric environment. Increased gastric pH and mucosal inflammation may reduce vitamin C concentration in gastric juice. At the same time, a less acidic environment impairs the release of vitamin B_12_ from dietary proteins, which may disrupt its absorption. As a result, *H. pylori* infection may indirectly influence iron bioavailability by reducing vitamin C availability, which supports its absorption, and by impairing vitamin B_12_ absorption. However, these mechanisms differ in nature and represent independent consequences of infection-induced changes in gastric physiology. Beyond its role in iron metabolism, vitamin C may also influence gastric physiology and *H. pylori* biology through antioxidant, mucosal-protective, and potential antimicrobial mechanisms, which together may contribute to modulation of the gastric microenvironment. Therefore, future studies are needed to determine whether modulation of the gastric environment by vitamin C translates into improved iron status and indirectly supports vitamin B_12_ availability in patients with *H. pylori* infection.

## Figures and Tables

**Table 3:** Effects of vitamin C supplementation on *Helicobacter pylori* infection and eradication.

Study Design/Population	Vitamin C Supplementation	Eradication Therapy	Main Findings	Ref.
Interventional clinical study; patients with *H. pylori*-positive non-ulcer dyspepsia (*n* = 30)	Vitamin C 500 mg twice daily + vitamin E 200 IU twice daily for 4 weeks	-	Increased gastric mucosal vitamin levels and decreased *H. pylori* density and neutrophil activity in the antrum	[59]
Randomized trial; patients with *H. pylori* infection (*n* = 160)	Vitamin C 500 mg twice daily + vitamin E 200 IU twice daily for 30 days	Lansoprazole + amoxicillin + clarithromycin + bismuth subcitrate	Higher eradication rate with antioxidant supplementation (91.25% vs. 60%)	[61]
Randomized trial; patients with *H. pylori* infection (*n* = 200)	Vitamin C 500 mg twice daily + vitamin E 200 IU twice daily for 30 days	Lansoprazole + amoxicillin + clarithromycin	Higher eradication rate with vitamin supplementation (82.5% vs. 45%)	[62]
Randomized double-blind controlled trial; adults with *H. pylori* gastritis (*n* = 117)	Vitamin C 200 mg twice daily + vitamin E 50 mg twice daily for 4 weeks	Bismuth + tetracycline + metronidazole (triple therapy) or placebo	No significant effect of vitamin C and E supplementation	[63]
Randomized controlled clinical trial; patients with *H. pylori* infection (*n* = 312)	Vitamin C 500 mg/day for 2 weeks	Amoxicillin + metronidazole + bismuth + omeprazole	Higher eradication rate with antioxidant supplementation (78% vs. 48.8%)	[64]
Randomized double-blind placebo-controlled study; patients with *H. pylori* infection (*n* = 38)	Vitamin C 5 g/day for 28 days	-	No significant effect of vitamin C supplementation	[65]
Randomized controlled clinical trial; *H. pylori*-infected patients (*n* = 104)	Vitamin C 250 mg twice daily + vitamin E 200 mg twice daily for 1 week, followed by vitamin C 250 mg/day and vitamin E 200 mg/day for 6 weeks	Lansoprazole + amoxicillin + metronidazole	No significant effect of vitamin C and E supplementation. Addition of vitamins C and E may even reduce eradication efficacy in patients infected with metronidazole-susceptible strains.	[66]
Randomized controlled clinical trial; adults with *H. pylori* infection (*n* = 90)	Vitamin C 500 mg three times daily for 7 days	Pantoprazole + amoxicillin + clarithromycin	No significant effect of vitamin C supplementation	[67]

## Data Availability

No new data were created or analyzed in this study.

## Abbreviations

The following abbreviations are used in this manuscript:

CagA	Cytotoxin-associated gene A
DHA	Dehydroascorbic acid
Fur	Ferric Uptake Regulator
gGT	Gamma-glutamyl transpeptidase
GSH	Glutathione
Hb	Hemoglobin
HP-NAP	Neutrophil-activating protein
IDA	Iron-deficiency anemia
IF	Intrinsic factor
LPS	Lipopolysaccharide
MCV	Mean corpuscular volume
PPI	Proton pump inhibitor
ROS	Reactive oxygen species
SVCT	Sodium-ascorbate cotransporters
VacA	Vacuolating cytotoxin
VBNC	Viable-but-non-culturable

## References

[1] Deng J., Ramelli L., Li P.Y., Eshaghpour A., Li A., Schuenemann G., Crowther M.A. (2024). Efficacy of Vitamin C with Fe Supplementation in Patients with Iron Deficiency Anemia: A Systematic Review and Meta-Analysis. Blood Vessel. Thromb. Hemost..

[2] Ukey U.U., Sharma S.K., Chitre D.S., Waghmare P.R., Dabir A.J., Desai R., Palatty N. (2024). Effect of Oral Vitamin C Administration along with Iron Supplementation for Treating Anaemia among Adolescent Girls—Protocol for Systematic Review and Meta—Analysis. J. Fam. Med. Prim. Care.

[3] Hou B., Zhang M., Liu M., Dai W., Lin Y., Li Y., Gong M., Wang G. (2019). Association of Active *Helicobacter pylori* Infection and Anemia in Elderly Males. BMC Infect. Dis..

[4] Haile K., Yemane T., Tesfaye G., Wolde D., Timerga A., Haile A. (2021). Anemia and Its Association with *Helicobacter pylori* Infection among Adult Dyspeptic Patients Attending Wachemo University Nigist Eleni Mohammad Memorial Referral Hospital, Southwest Ethiopia: A Cross-Sectional Study. PLoS ONE.

[5] Nguyen R.N., Bui N.Q., Nguyen K.O.T. (2024). Prevalence, Predictors, and Treatment Outcomes of Anemia in Vietnamese School-Age Children with *Helicobacter pylori* Infection. Cureus.

[6] Senkovich O., Ceaser S., McGee D.J., Testerman T.L. (2010). Unique Host Iron Utilization Mechanisms of *Helicobacter pylori* Revealed with Iron-Deficient Chemically Defined Media. Infect. Immun..

[7] El Demerdash D.M., Ibrahim H., Hassan D.M., Moustafa H., Tawfik N.M. (2018). *Helicobacter pylori* Associated to Unexplained or Refractory Iron Deficiency Anemia: An Egyptian Single-Center Experience. Hematol. Transfus. Cell Ther..

[8] Elamin E.A.I., Suliman M.A., Azoz M.E., Ali E.W., Olerile L.D., Jiao Y., Zhao Y. (2018). Effect of *Helicobacter pylori* Infection on Haematological Parameters in Kosti Teaching Hospital, Sudan. Iran. Red Crescent Med. J..

[9] Pustelnik E., Pikora K., Hartman M., Kurzeja M., Czuwara J., Łaguna P. (2025). Multifaceted Clinical Spectrum of Vitamin B12 Deficiency—A Case Report and Literature Review. J. Blood Med..

[10] Van Vranken M. (2010). Evaluation of Microcytosis. Am. Fam. Physician.

[11] Basrowi R.W., Dilantika C. (2021). Optimizing Iron Adequacy and Absorption to Prevent Iron Deficiency Anemia: The Role of Combination of Fortified Iron and Vitamin C. World Nutr. J..

[12] Toh J.W.T., Wilson R.B. (2020). Pathways of Gastric Carcinogenesis, *Helicobacter pylori* Virulence and Interactions with Antioxidant Systems, Vitamin C and Phytochemicals. Int. J. Mol. Sci..

[13] Temova Rakuša Ž., Roškar R., Hickey N., Geremia S. (2022). Vitamin B12 in Foods, Food Supplements, and Medicines—A Review of Its Role and Properties with a Focus on Its Stability. Molecules.

[14] Meščić Macan A., Gazivoda Kraljević T., Raić-Malić S. (2019). Therapeutic Perspective of Vitamin C and Its Derivatives. Antioxidants.

[15] Bhoot H.R., Zamwar U.M., Chakole S., Anjankar A. (2023). Dietary Sources, Bioavailability, and Functions of Ascorbic Acid (Vitamin C) and Its Role in the Common Cold, Tissue Healing, and Iron Metabolism. Cureus.

[16] Foyer C.H., Kunert K. (2024). The Ascorbate–Glutathione Cycle Coming of Age. J. Exp. Bot..

[17] Yin X., Chen K., Cheng H., Chen X., Feng S., Song Y., Liang L. (2022). Chemical Stability of Ascorbic Acid Integrated into Commercial Products: A Review on Bioactivity and Delivery Technology. Antioxidants.

[18] Doseděl M., Jirkovský E., Macáková K., Krčmová L., Javorská L., Pourová J., Mercolini L., Remião F., Nováková L., Mladěnka P. (2021). Vitamin C—Sources, Physiological Role, Kinetics, Deficiency, Use, Toxicity, and Determination. Nutrients.

[19] Akbari A., Jelodar G., Nazifi S., Sajedianfard J. (2016). An Overview of the Characteristics and Function of Vitamin C in Various Tissues: Relying on Its Antioxidant Function. Zahedan J. Res. Med. Sci..

[20] Aditi A., Graham D.Y. (2012). Vitamin C, Gastritis, and Gastric Disease: A Historical Review and Update. Dig. Dis. Sci..

[21] Sobala G., Schorah C., Sanderson M., Dixon M., Tompkins D., Godwin P., Axon A.T.R. (1989). Ascorbic Acid in the Human Stomach. Gastroenterology.

[22] Carr A., Maggini S. (2017). Vitamin C and Immune Function. Nutrients.

[23] Rychlik E., Stoś K., Woźniak A., Mojska H. (2024). Normy Żywienia Dla Populacji Polski.

[24] National Institutes of Health Office of Dietary Supplements. Vitamin C. Fact Sheet for Health Professionals. https://ods.od.nih.gov/factsheets/VitaminC-HealthProfessional/.

[25] Hurrell R.F. (2022). Ensuring the Efficacious Iron Fortification of Foods: A Tale of Two Barriers. Nutrients.

[26] Li N., Zhao G., Wu W., Zhang M., Liu W., Chen Q., Wang X. (2020). The Efficacy and Safety of Vitamin C for Iron Supplementation in Adult Patients with Iron Deficiency Anemia. JAMA Netw. Open.

[27] Khoshfetrat M.R., Mortazavi S., Neyestani T., Mahmoodi M.R., Zerafati-Shoae N., Mohammadi-Nasrabadi F. (2014). Iron and Vitamin C Co-Supplementation Increased Serum Vitamin C without Adverse Effect on Zinc Level in Iron Deficient Female Youth. Int. J. Prev. Med..

[28] Dwi Astuti N., Wirjatmadi B., Adriani M. (2018). The Role of Addition of Vitamin C in Iron Supplementation on Ferritin Serum Levels in Anemia Adolescent Females. Health Notions.

[29] Ahmad I., Qadeer K., Zahid S., Sheraz M.A., Ismail T., Hussain W., Ansari I.A. (2014). Effect of Ascorbic Acid on the Degradation of Cyanocobalamin and Hydroxocobalamin in Aqueous Solution: A Kinetic Study. AAPS PharmSciTech.

[30] Schnellbaecher A., Binder D., Bellmaine S., Zimmer A. (2019). Vitamins in Cell Culture Media: Stability and Stabilization Strategies. Biotechnol. Bioeng..

[31] Simon J.A., Hudes E.S. (1999). Relation of Serum Ascorbic Acid to Serum Vitamin B12, Serum Ferritin, and Kidney Stones in US Adults. Arch. Intern. Med..

[32] Balabanova L., Averianova L., Marchenok M., Son O., Tekutyeva L. (2021). Microbial and Genetic Resources for Cobalamin (Vitamin B12) Biosynthesis: From Ecosystems to Industrial Biotechnology. Int. J. Mol. Sci..

[33] Watanabe F. (2007). Vitamin B 12 Sources and Bioavailability. Exp. Biol. Med..

[34] Chamlagain B., Sugito T.A., Deptula P., Edelmann M., Kariluoto S., Varmanen P., Piironen V. (2018). In Situ Production of Active Vitamin B12 in Cereal Matrices Using *Propionibacterium freudenreichii*. Food Sci. Nutr..

[35] National Institutes of Health. Office of Dietary Supplements Vitamin B12. Fact Sheet for Health Professionals. https://ods.od.nih.gov/factsheets/VitaminB12-HealthProfessional/.

[36] Ermens A.A.M., Vlasveld L.T., Lindemans J. (2003). Significance of Elevated Cobalamin (Vitamin B12) Levels in Blood. Clin. Biochem..

[37] Pushpakumar S., Kundu S., Sen U. (2014). Endothelial Dysfunction: The Link Between Homocysteine and Hydrogen Sulfide. Curr. Med. Chem..

[38] Peng X., Gao Q., Zhou J., Ma J., Zhao D., Hao L. (2021). Association between Dietary Antioxidant Vitamins Intake and Homocysteine Levels in Middle-Aged and Older Adults with Hypertension: A Cross-Sectional Study. BMJ Open.

[39] Kumari S., Selvakumar C. (2024). Prevalence and Clinical Hematological Profile of Vitamin B12 Deficiency Associated Megaloblastic Anemia in Children: A Hospital-Based Observational Study. Int. J. Pharm. Clin. Res..

[40] Singh R.S.A.K., Manikanta V., Tallada T.B. (2025). Prevalence and Clinico-Hematological Profile of Megaloblastic Anemia in Children Aged 1–14 Years: A Hospital-Based Study. Eur. J. Cardiovasc. Med..

[41] Liu Y., Miao R., Xia J., Zhou Y., Yao J., Shao S. (2024). Infection of *Helicobacter pylori* Contributes to the Progression of Gastric Cancer through Ferroptosis. Cell Death Discov..

[42] Gowdappa H.B., Mahesh M., Murthy K.V.K.S.N., Narahari M.G. (2013). *Helicobacter pylori* Associated Vitamin B 12 Deficiency, Pernicious Anaemia and Subacute Combined Degeneration of the Spinal Cord. BMJ Case Rep..

[43] Bravo D., Hoare A., Soto C., Valenzuela M.A., Quest A.F. (2018). *Helicobacter pylori* in Human Health and Disease: Mechanisms for Local Gastric and Systemic Effects. World J. Gastroenterol..

[44] Motupalli S.K., Oroszi T.L. (2024). The Nexus between *Helicobacter pylori* Infection and Anemia—A Systematic Review. Front. Hematol..

[45] Zamani M., Vahedi A., Maghdouri Z., Shokri-Shirvani J. (2017). Role of Food in Environmental Transmission of *Helicobacter pylori*. Casp. J. Intern. Med..

[46] Bińkowska A., Biernat M., Duś I., Gościniak G. (2013). The Role of Biofilm Formation in Pathogenesis of *Helicobacter pylori* Infections. Gastroenterol. Rev..

[47] Quaglia N.C., Dambrosio A. (2018). *Helicobacter pylori*: A Foodborne Pathogen?. World J. Gastroenterol..

[48] Almashhadany D.A., Zainel M.A., AbdulRahman T.T. (2024). Review of Foodborne Helicobacteriosis. Ital. J. Food Saf..

[49] Lina T.T., Alzahrani S., Gonzalez J., Pinchuk I.V., Beswick E.J., Reyes V.E. (2014). Immune Evasion Strategies Used by *Helicobacter pylori*. World J. Gastroenterol..

[50] Zheng P.Y., Jones N.L. (2003). *Helicobacter pylori* Strains Expressing the Vacuolating Cytotoxin Interrupt Phagosome Maturation in Macrophages by Recruiting and Retaining TACO (Coronin 1) Protein. Cell. Microbiol..

[51] Tegtmeyer N., Wessler S., Backert S. (2011). Role of the Cag -pathogenicity Island Encoded Type IV Secretion System in *Helicobacter pylori* Pathogenesis. FEBS J..

[52] Hussain A., Tabrez E., Peela J.R., Honnavar P., Tabrez S.S.M. (2018). Vitamin C: A Preventative, Therapeutic Agent Against *Helicobacter pylori*. Cureus.

[53] Ansari S., Yamaoka Y. (2017). Survival of *Helicobacter pylori* in Gastric Acidic Territory. Helicobacter.

[54] Di Fermo P., Di Lodovico S., Di Campli E., D’Arcangelo S., Diban F., D’Ercole S., Di Giulio M., Cellini L. (2023). *Helicobacter pylori* Dormant States Are Affected by Vitamin C. Int. J. Mol. Sci..

[55] Krzyżek P., Gościniak G. (2018). Morphology of *Helicobacter pylori* as a Result of Peptidoglycan and Cytoskeleton Rearrangements. Gastroenterol. Rev..

[56] Hathroubi S., Zerebinski J., Ottemann K.M. (2018). *Helicobacter pylori* Biofilm Involves a Multigene Stress-Biased Response, Including a Structural Role for Flagella. mBio.

[57] Van Vliet A.H.M., Stoof J., Vlasblom R., Wainwright S.A., Hughes N.J., Kelly D.J., Bereswill S., Bijlsma J.J.E., Hoogenboezem T., Vandenbroucke-Grauls C.M.J.E. (2002). The Role of the Ferric Uptake Regulator (Fur) in Regulation of *Helicobacter pylori* Iron Uptake. Helicobacter.

[58] Pich O.Q., Merrell D.S. (2013). The Ferric Uptake Regulator of *Helicobacter pylori*: A Critical Player in the Battle for Iron and Colonization of the Stomach. Future Microbiol..

[59] Sezikli M., Çetinkaya Z.A., Güzelbulut F., Çimen B., Özcan Ö., Özkara S., Yeşil A., Gümrükçü G., İpçioğlu O.M., Sezikli H. (2012). Effects of Alpha Tocopherol and Ascorbic Acid on *Helicobacter pylori* Colonization and the Severity of Gastric Inflammation. Helicobacter.

[60] Naja F., Kreiger N., Mckeown Eyssen G., Allard J. (2010). Bioavailability of Vitamins E and C: Does *Helicobacter pylori* Infection Play a Role?. Ann. Nutr. Metab..

[61] Sezikli M., Çetinkaya Z.A., Sezikli H., Güzelbulut F., Tiftikçi A., Tüzün İnce A., Gökden Y., Yaşar B., Atalay S., Övünç Kurdaş O. (2009). Oxidative Stress in *Helicobacter pylori* Infection: Does Supplementation with Vitamins C and E Increase the Eradication Rate?. Helicobacter.

[62] Sezikli M., Çetinkaya Z.A., Güzelbulut F., Yeşil A., Coşgun S., Kurdaş O.Ö (2012). Supplementing Vitamins C and E to Standard Triple Therapy for the Eradication of *Helicobacter pylori*. J. Clin. Pharm. Ther..

[63] Everett S.M., Drake I.M., White K.L.M., Mapstone N.P., Chalmers D.M., Schorah C.J., Axon A.T.R. (2002). Antioxidant Vitamin Supplements Do Not Reduce Reactive Oxygen Species Activity in *Helicobacter pylori* Gastritis in the Short Term. Br. J. Nutr..

[64] Zojaji H., Talaie R., Mirsattari D., Haghazali M., Molaei M., Mohsenian N., Derakhshan F., Zali M.R. (2009). The Efficacy of *Helicobacter pylori* Eradication Regimen with and without Vitamin C Supplementation. Dig. Liver Dis..

[65] Kamiji M.M., Oliveira R.B. (2005). de Efeito Da Administração de Vitamina C Sobre a Colonização Do Estômago Por *Helicobacter pylori*. Arq. Gastroenterol..

[66] Chuang C., Sheu B., Huang A., Yang H., Wu J. (2002). Vitamin C and E Supplements to Lansoprazole-Amoxicillin-Metronidazole Triple Therapy May Reduce the Eradication Rate of Metronidazole—Susceptible *Helicobacter pylori* Infection. Helicobacter.

[67] Namiot A., Namiot D., Bucki R., Kemona A., Kurylonek A., Namiot Z. (2020). Vitamin C Does Not Improve the Efficacy of *Helicobacter pylori* Eradication in Smokers. Prog. Health Sci..

[68] Kato S., Gold B.D., Kato A. (2022). *Helicobacter pylori*-Associated Iron Deficiency Anemia in Childhood and Adolescence-Pathogenesis and Clinical Management Strategy. J. Clin. Med..

[69] Annibale B., Capurso G., Lahner E., Passi S., Ricci R., Maggio F., Delle Fave G. (2003). Concomitant Alterations in Intragastric PH and Ascorbic Acid Concentration in Patients with *Helicobacter pylori* Gastritis and Associated Iron Deficiency Anaemia. Gut.

[70] Sasazuki S., Sasaki S., Tsubono Y., Okubo S., Hayashi M., Kakizoe T., Tsugane S. (2003). The Effect of 5-year Vitamin C Supplementation on Serum Pepsinogen Level and *Helicobacter pylori* Infection. Cancer Sci..

[71] Sung J., Kim N., Lee J., Hwang Y.-J., Kim H.W., Chung J.W., Kim J.-W., Lee D.H. (2018). Associations among Gastric Juice PH, Atrophic Gastritis, Intestinal Metaplasia and *Helicobacter pylori* Infection. Gut Liver.

[72] Safranow K., Korzonek M., Dziedziejko V., Jakubowska K., Sulzyc-Bielicka V., Domański L., Ciechanowski K., Chlubek D. (2006). Influence of Gastric Juice PH on the Metabolism of Vitamin C in Gastric Mucosa and Juice. Pol. Merkur. Lek. Organ Pol. Tow. Lek..

[73] Phull P.S., Price A.B., White K.L.M., Schorah C.J., Jacyna M.R. (1999). Gastroduodenal Mucosal Vitamin-C Levels in *Helicobacter pylori* Infection. Scand. J. Gastroenterol..

[74] Zhang Z.W., Patchett S.E., Perrett D., Katelaris P.H., Domizio P., Farthing M.J.G. (1998). The Relation between Gastric Vitamin C Concentrations, Mucosal Histology, and CagA Seropositivity in the Human Stomach. Gut.

[75] Capurso G., Ricci R., Panzuto F., Baccini F., Passi S., Di Giulio E., Fave G.D., Annibale B. (2003). Intragastric Ascorbic But Not Uric Acid Is Depleted in Relation with the Increased PH in Patients with Atrophic Body Gastritis and *H. pylori* Gastritis. Helicobacter.

[76] Kodama K., Sumii K., Kawano M., Kido T., Nojima K., Sumii M., Haruma K., Yoshihara M., Chayama K. (2003). Gastric Juice Nitrite and Vitamin C in Patients with Gastric Cancer and Atrophic Gastritis. Eur. J. Gastroenterol. Hepatol..

[77] Zhang Z.W., Farthing M.J. (2005). The Roles of Vitamin C in *Helicobacter pylori* Associated Gastric Carcinogenesis. Chin. J. Dig. Dis..

[78] Annibale B., Capurso G., Delle Fave G. (2002). Consequences of *Helicobacter pylori* Infection on the Absorption of Micronutrients. Dig. Liver Dis..

[79] O’Leary F., Samman S. (2010). Vitamin B12 in Health and Disease. Nutrients.

[80] Nabavi-Rad A., Azizi M., Jamshidizadeh S., Sadeghi A., Aghdaei H.A., Yadegar A., Zali M.R. (2022). The Effects of Vitamins and Micronutrients on *Helicobacter pylori* Pathogenicity, Survival, and Eradication: A Crosstalk between Micronutrients and Immune System. J. Immunol. Res..

[81] Cohen H. (2000). Heterogeneity of Gastric Histology and Function in Food Cobalamin Malabsorption: Absence of Atrophic Gastritis and Achlorhydria in Some Patients with Severe Malabsorption. Gut.

[82] Serin E., Gümürdülü Y., Özer B., Kayaselçuk F., Yilmaz U., Koçak R. (2002). Impact of *Helicobacter pylori* on the Development of Vitamin B 12 Deficiency in the Absence of Gastric Atrophy. Helicobacter.

[83] Kaptan K., Beyan C., Ural A.U., Çetin T., Avcu F., Gülşen M., Finci R., Yalçín A. (2000). *Helicobacter pylori*—Is It a Novel Causative Agent in Vitamin B12 Deficiency?. Arch. Intern. Med..

[84] Lewerin C., Jacobsson S., Lindstedt G., Nilsson-Ehle H. (2008). Serum Biomarkers for Atrophic Gastritis and Antibodies against *Helicobacter pylori* in the Elderly: Implications for Vitamin B 12, Folic Acid and Iron Status and Response to Oral Vitamin Therapy. Scand. J. Gastroenterol..

[85] Pérez Roldán F., Castellanos Monedero J.J., González Carro P., Villafáñez García M.C., Roncero García-Escribano Ó., Legaz Huidobro M.L., Ruiz Carrillo F. (2008). Efecto de La Erradicación de *Helicobacter pylori* En La Anemia Ferropénica de Origen Incierto. Gastroenterol. Y Hepatol..

[86] Vieira R.R., Fontes L.E.S., Pacheco R.L., Fernandes M.A.P., Malta P.P., Riera R. (2020). Proton Pump Inhibitor- and Clarithromycin-Based Triple Therapies for *Helicobacter pylori* Eradication. Cochrane Database Syst. Rev..

[87] Kim S.Y., Chung J.-W. (2020). Best *Helicobacter pylori* Eradication Strategy in the Era of Antibiotic Resistance. Antibiotics.

[88] McColl K.E.L. (2009). Effect of Proton Pump Inhibitors on Vitamins and Iron. Am. J. Gastroenterol..

[89] Mumtaz H., Ghafoor B., Saghir H., Tariq M., Dahar K., Ali S.H., Waheed S.T., Syed A.A. (2022). Association of Vitamin B12 Deficiency with Long-Term PPIs Use: A Cohort Study. Ann. Med. Surg..

[90] Sarzynski E., Puttarajappa C., Xie Y., Grover M., Laird-Fick H. (2011). Association Between Proton Pump Inhibitor Use and Anemia: A Retrospective Cohort Study. Dig. Dis. Sci..

[91] McCarthy D. (2019). Iron Deficiency Anaemia Due to Proton Pump Inhibitors: Clinical Impact Revealed. J. Intern. Med..

[92] Losurdo G., Caccavo N.L.B., Indellicati G., Celiberto F., Ierardi E., Barone M., Di Leo A. (2023). Effect of Long-Term Proton Pump Inhibitor Use on Blood Vitamins and Minerals: A Primary Care Setting Study. J. Clin. Med..

[93] Jiang X., Doyle M.P. (2000). Growth Supplements for *Helicobacter pylori*. J. Clin. Microbiol..

[94] Mahalanabis D., Islam M.A., Shaikh S., Chakrabarty M., Kurpad A.V., Mukherjee S., Sen B., Khaled M.A., Vermund S.H. (2005). Haematological Response to Iron Supplementation Is Reduced in Children with Asymptomatic *Helicobacter pylori* Infection. Br. J. Nutr..

[95] Rajendiran S., Zachariah B., Hamide A. (2012). Increased Protein Carbonylation and Decreased Antioxidant Status in Anemic *H. Pylori* Infected Patients: Effect of Treatment. Saudi J. Gastroenterol..

[96] Duque X., Moran S., Mera R., Medina M., Martinez H., Mendoza M.E., Torres J., Correa P. (2010). Effect of Eradication of *Helicobacter pylori* and Iron Supplementation on the Iron Status of Children with Iron Deficiency. Arch. Med. Res..

[97] Sarker S.A., Mahmud H., Davidsson L., Alam N.H., Ahmed T., Alam N., Salam M.A., Beglinger C., Gyr N., Fuchs G.J. (2008). Causal Relationship of *Helicobacter pylori* with Iron-Deficiency Anemia or Failure of Iron Supplementation in Children. Gastroenterology.

[98] Beckett A.C., Piazuelo M.B., Noto J.M., Peek R.M., Washington M.K., Algood H.M.S., Cover T.L. (2016). Dietary Composition Influences Incidence of *Helicobacter pylori*-Induced Iron Deficiency Anemia and Gastric Ulceration. Infect. Immun..

